# Ptychoscopy: a user friendly experimental design tool for ptychography

**DOI:** 10.1038/s41598-025-09871-6

**Published:** 2025-07-10

**Authors:** Radim Skoupy, Elisabeth Müller, Timothy J. Pennycook, Manuel Guizar-Sicairos, Emiliana Fabbri, Emiliya Poghosyan

**Affiliations:** 1 PSI Center for Life Sciences, Paul Scherrer Institute, PSI, Villigen, 5232 Switzerland; 2 EMAT, Department of Physics, University of Antwerp, Groenenborgerlaan 171, Antwerp, 2020 Belgium; 3 PSI Center for Photon Science, Paul Scherrer Institute, PSI, Villigen, 5232 Switzerland; 4 École Polytechnique Fédérale de Lausanne, Lausanne, 1015 Switzerland; 5 PSI Center for Energy and Environmental Sciences, Paul Scherrer Institute, PSI, Villigen, 5232 Switzerland

**Keywords:** Electron ptychography, Experimental design, Real- and reciprocal space sampling, Transmission electron microscopy, Imaging and sensing, Electron microscopy

## Abstract

Electron ptychography is a rapidly growing diffractive imaging technique providing superior image contrast, high resolution, efficient dose usage and versatile imaging conditions. Originally coming from the physical sciences, it has also become a method of interest in the life sciences. Regardless of the scientific field, the successful ptychographic reconstruction relies on various combinations of adjustable experimental parameters, along with the choice of the reconstruction algorithm. In this work we present *ptychoScopy*, a Python-based tool developed to simplify the selection of these parameters and aid successful experimental design. Using a $\hbox {SmB}_{6}$ sample, we show the influence of key parameters such as probe convergence angle, defocus and electron dose on the reconstruction quality using direct and iterative reconstruction algorithms. We investigate the influence of real and reciprocal space sampling and their influence on the speed and quality of ptychographic reconstructions. PtychoScopy simplifies experimental design and guides the researchers across diverse scientific fields in setting up successful ptychographic experiments.

## Introduction

Various techniques have emerged providing scanning transmission electron microscopy (STEM) with enhanced ability to reveal light elements and improved overall dose efficiency compared to Z-contrast annular dark field (ADF) imaging. These include annular bright field (ABF)^1^ and the scattering centre-of-mass-based imaging techniques such as integrated differential phase contrast (iDPC^2^) and integrated centre of mass (iCOM). Ptychography^3^ has also seen a resurgence of interest in electron microscopy, offering very high dose efficiency, simultaneous sensitivity to heavy and light elements, the ability to recover the incident illumination, including its aberrations, and deconvolve its influence so that resolution much finer than that given by the illumination can be obtained^4–7^.

**Fig. 1:**
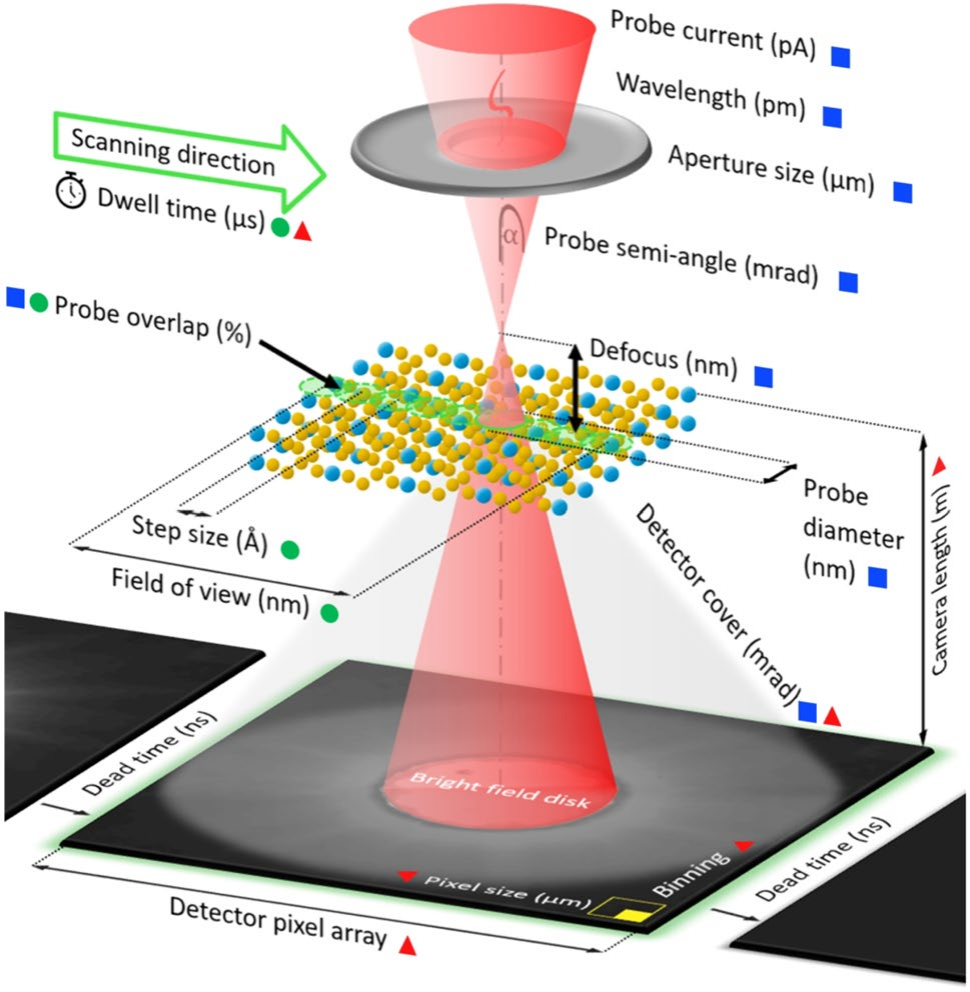
Adjustable parameters for ptychographic data acquisition and where to find them. Electron beam settings (blue filled squares), scanning parameters (light green filled circles) and detection characteristics (red filled triangles).

Recent advances in pixelated detector technologies have made 4D STEM methods such as ptychography much more practical for electron microscopy. These advances include direct electron detection and frame rates greater than 100 kHz as with the Dectris ARINA^8^, which is used in the present study, and event based nanosecond time resolution detectors such as the Timepix3^9,10^. High speed detectors reduce acquisition times, greatly alleviating sample drift and providing a route to lower dose scans that can prevent or reduce radiation damage. The flexibility of ptychography with respect to experimental parameters allows one to tailor the data acquisition process specifically to the material and scientific question, ranging from beam tolerant samples with extreme resolution^11,12^ to very sensitive biological specimens^13–15^. However optimal design of ptychographic experiments requires careful selection of acquisition parameters, as e.g. probe semi-angle, real- and reciprocal space sampling, and depends on which of the many ptychographic methods is preferred. Careful choice of the parameters, an overview of which is shown in Figure 1, can lead to an optimum balance of high-resolution and dose-efficiency which is especially important for a wide range of beam-sensitive samples.

Various studies so far probed the influence of combination of different parameters on the ptychographic reconstruction outcome, including probe semi-angle^14^; detector coverage angles together with dose^16^; single- and mixed state reconstructions related to electron dose^17^; real- and reciprocal space sampling for multislice reconstructions^18^; scan step size, detector size, defocus and beam semi-angle with respect to the electron dose^19^. However, non of them covered an extensive parameter space necessary for the flexible ptychographic experimental setup.

Direct ptychography methods, such as the single side band^20,21^ (SSB) and Wigner distribution deconvolution^22–24^ (WDD), retrieve the phase information from locations in reciprocal space using analytical expressions. Both direct methods require fine real space sampling, and although a degree of defocus can sometimes be beneficial for optimum contrast^10^, a focused or mostly focused probe is normally employed. Both SSB and WDD can also provide post-acquisition aberration correction^23,25^ and superresolution which is transferred by the interference of higher order beams, and can be determined by the process of stepping out in direct methods^22^. Even though WDD requires an extra Fourier transform compared to SSB and is therefore significantly slower, both methods are capable of live reconstruction^24,26,27^. However, with little if any practical benefits observed for WDD over SSB, we focus on SSB here. Nonetheless, very similar parameters to those optimal for SSB will also usually be optimal for WDD.

Iterative ptychography methods (ITR) rely on reconstructing the electron wave exiting the sample and its decomposition into a product of the probe and object using overlapping illumination footprints^28^. ITR methods provide superresolution by default when sufficient signal is present at angles higher than the bright field disk and can benefit from higher reciprocal space sampling. Furthermore, using ITR one can account for effects of sample thickness such as multiple scattering by using multislice methods^29^ in which a probe and object solution is sought at a set of slices of the sample along the beam direction rather than approximating the entire sample as a single thin object as in singleslice reconstructions. In addition, these multislice solutions can be used for 3D structure determination^30^.

The choice of a suitable ptychographic reconstruction algorithm depends on the sample type, radiation sensitivity, available hardware and the desired experimental outcome. An overview of existing reconstruction algorithms can be found in Supplementary Table S1. Due to the considerable influence of the method of choice on the experimental setup and in order to assist researchers in selecting optimal experimental parameters and suitable reconstruction techniques, we have developed *ptychoScopy*^31^, a Python tool that simplifies and guides the design and execution of electron ptychography experiments. Using our tool, we demonstrate the impact of optimal versus suboptimal parameter selection with examples using experimental $\hbox {SmB}_{6}$ data, containing both, light and heavy atoms. In addition, we discuss the influence of real- and reciprocal space sampling on the data acquisition quality comparing the performance of SSB and ITR methods.

## Results

### PtychoScopy

**Fig. 2:**
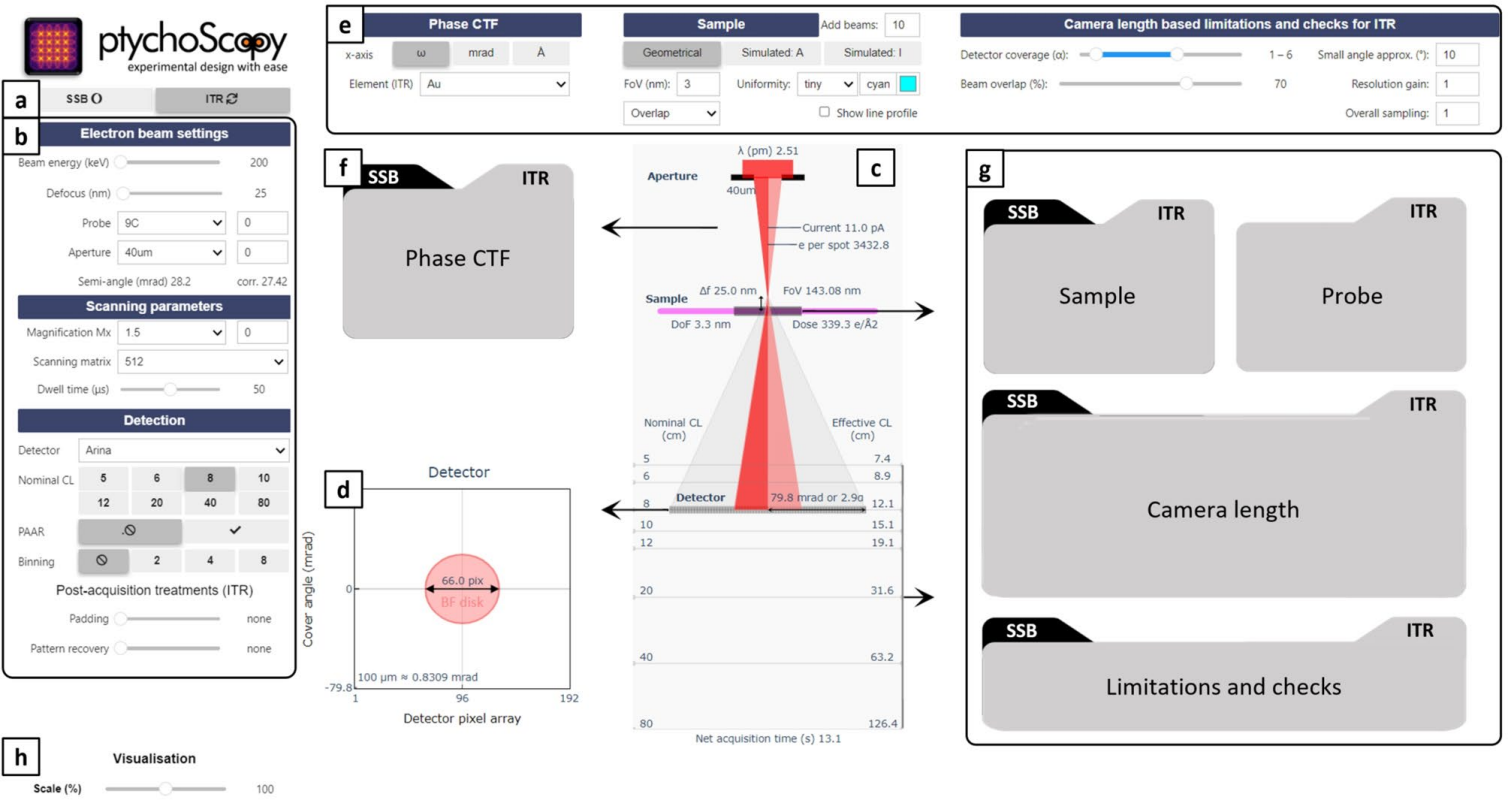
PtychoScopy main panel. (**a**) Selection of the reconstruction method. (**b**) Microscope control panel divided into parts for *Electron beam settings*, *Scanning parameters* and *Detection* respectively. (**c**) Microscope sketch drawing. (**d**) Detector scheme. (**e**) Graph control for *Phase CTF*, *Sample* plane and *Camera length based limitations and checks*. (**f**, **g**) The gray areas are reconstruction method related charts for phase CTF (**f**) and for sample plane—camera length (**g**) respectively. (**h**) Graph size control.

*PtychoScopy* provides an interactive GUI through which the user can examine the effect of choosing different experimental parameters with different ptychographic algorithms for a specific microscope setup as shown in Fig. 2.

*PtychoScopy* simplifies the setup of ptychographic experiments by incorporating microscope specific calibration data as loaded from a customizable *calibrations.xlsx* file containing the essential parameters available on a given setup, including beam energies, magnifications, convergence semi-angles, camera lengths, and detector properties such as pixel size in both real- and reciprocal space. The parameters specified in the *calibrations.xlsx* are then provided in dropdown menus or other suitable interactive controls in the GUI (Fig. 2a, b). These can be set up and named according to, for instance, the lens settings and apertures used on a given microscope. The tool automatically generates a microscope sketch drawing (Fig. 2c) where information about the electron wavelength, probe current, field of view, applied electron dose, number of electrons per diffraction pattern, camera length, detection range in angle units and multiples of the beam semi-angle ($\alpha$) can be found. The detector plane is then shown in drawing Fig. 2d with information about the bright-field (BF) disk diameter in pixels (including binning) and the relative and absolute sizes of the detector pixels.

In addition, the first method-dependent block (Fig. 2f) contains the method-related phase contrast transfer function (CTF) controlled by Fig. 2e. The second block (Fig. 2g) displays information related to the real- and reciprocal space sampling, illumination uniformity, probe window and a guided-camera-length selection tool utilizing the built-in limitations and checks listed in Table 1 as configured through Fig. 2e. Graph size can then be controlled in Fig. 2h. Further detailed description of the tool controls can be found in Fig. S1.

**Table 1:** PtychoScopy built-in limitations and checks.

	Limitation/check	Description	Default
ITR	Detector coverage	Detector cover is at least BF disk and less than low count area.	1-6$\alpha$
	Probe overlap	Proper reconstruction requires significant real-space probe overlap.	70 %
	Small angle approx.	Limit suggests potential issues with unequal angular sampling.	$10^{\circ }$
	Resolution gain	Reconstructed image has more pixels than scanning beam positions.	1
	Combined sampling	Combined real- and reciprocal sampling is above the set level.	1
	Pattern recovery	Chosen pattern recovery level is lower than combined sampling.	Equation (7)
	Probe window coverage	Electron probe fulfills Nyquist sampling condition.	Equation (1)
SSB	Real-space sampling	Scanning step size is lower than probe semi-angle related optimal sampling.	Equation (3)
	Detector coverage	Detector cover is at least BF disk.	Fixed 1$\alpha$
	Minimal BF disk	BF disk diameter is larger than minimal limit found in Section Detector array size and its impact on reciprocal space sampling.	Fixed 4 pix

**Fig. 3:**
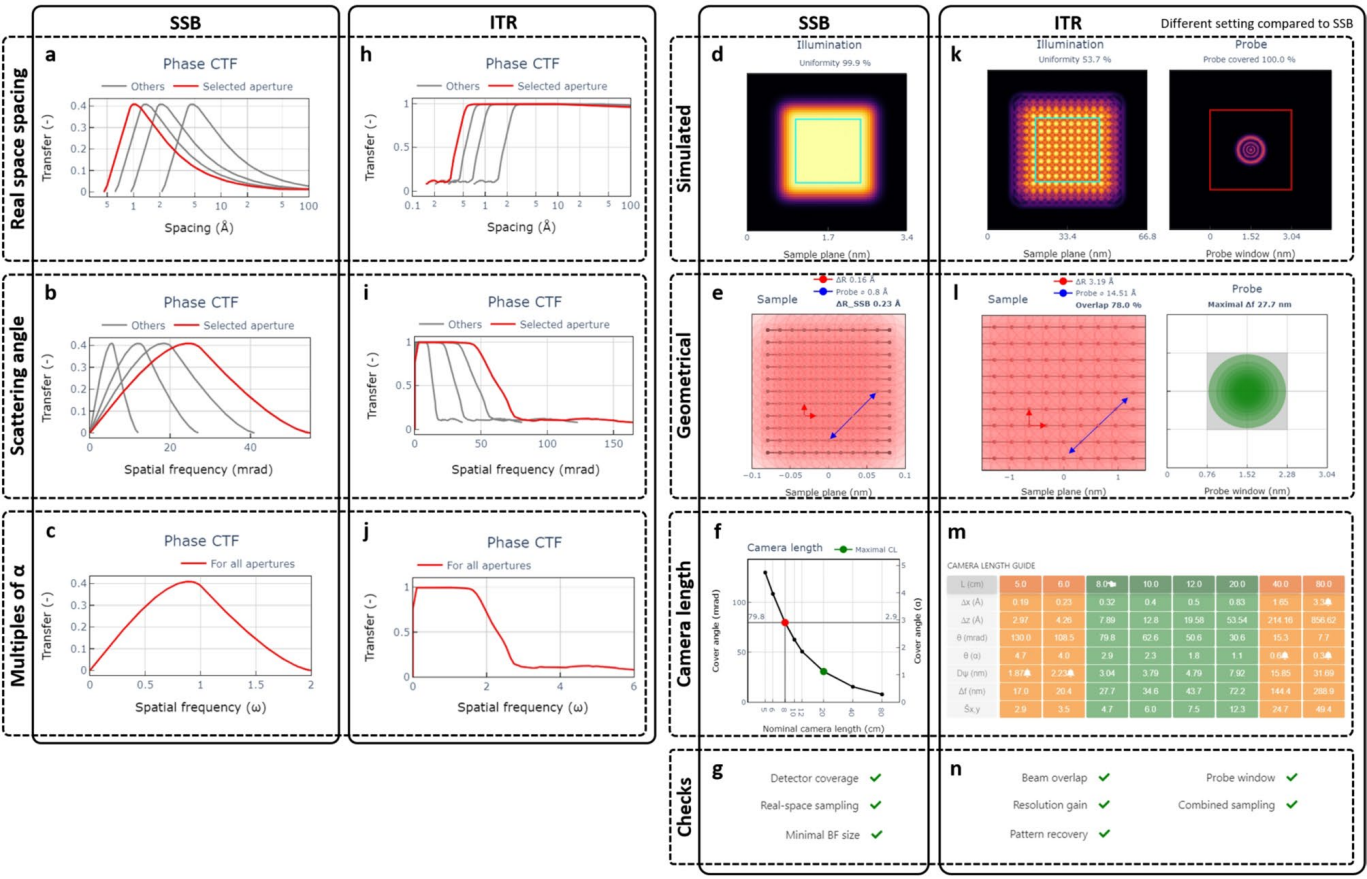
Reconstruction method related blocks. The CTF can for both, SSB (**a**–**c**) and ITR methods (**h**–**j**) be switched from multiplications of probe semi-angle to scattering angles or real space sampling. For ITR the sample plane charts dealing with real space probe density, corresponding beam overlap and related probe window can be found in geometrical (**l**) and simulated probe representation (**k**) in form of amplitude or intensity. Note: the geometrical beam diameter is computed from probe semi-angle, defocus and beam diameter in focus. The camera length guide (**m**) shows the important characteristics (reconstruction pixel size, minimal thickness of a slice for multislice reconstruction, detector coverage, probe window and overall sampling) with respect to nominal camera length and set checks which results can be found in (**n**). There is also a simplified version for SSB consisting of a sample plane chart (**d**, **e**) with recommended scanning step size (based on probe semi-angle related CTF) and maximum camera length enabling at least $1\alpha$ on detector (**f**). Results of checks can be found in (**g**).

Examples of the content of the reconstruction method related blocks are shown in Fig. 3. For SSB the intrinsic phase CTF is plotted in Fig. 3a–c , d﻿–e shows the beam position distribution with an optimal scanning step size recommendation (“Sample”), adjusted via Fig. 2e, and a camera length guide (“Camera length”) showing the maximum recommended value (Fig. 3f). Built-in checks are then shown in Fig. 3g. The ITR tab provides a numerical phase CTF (Fig. 3h–j) and a beam position visualisation, but also a probe window (“Probe”) plot that is important to consider for defocused probe ptychography (Fig. 3k, l) and a detailed chart to guide setting a proper camera length (Fig. 3m) including built-in checks (Fig. 3n). The probe window and the beam position chart can be switched from a simple geometrical representation into either intensity or amplitude maps using the beam shape simulated by the abTEM package^32^.

### Sample driven probe semi-angle selection

When designing an experiment one should consider which spatial frequencies are sought and choose parameters that weight their transfer accordingly. The CTF profile describes the transfer of spatial frequencies $Q_{p}$, whose shape is linked to the probe convergence semi-angle as $Q_{p} = \omega \alpha$ where $\omega$ is dimensionless number^33^.

The CTF for iterative methods is not well defined from first principles, but can be numerically computed with single atom simulations^34^. For simplicity the ITR CTFs included in *ptychoScopy* are calculated using a single atom of either C, Cu or Au and a focused probe. In Fig. 3h–j we present the ITR CTF curves calculated using an Au atom. The procedure is described in Methods. The ITR transfer functions increase rapidly from zero frequency to a plateau extending a substantial way out to $2\alpha$ ($\omega = 2$) and then decay with a tail that extends beyond $2\alpha$ due to superresolution. The simulation based ITR CTFs display some variation with respect to the atomic element used as heavier elements scatter electrons to higher angles more often. This makes sense as the nature of the diffraction pattern depends on the sample, including the high-angle disk overlaps that contribute to superresolution. Nevertheless the shapes are qualitatively quite similar.

The intrinsic SSB CTF is determined via first principles from the size of the overlap between the direct and diffracted beams in probe reciprocal space, excluding triple overlaps. These double disk overlap areas decrease in size from the maximum at $0.9\alpha$ in both the high- and low frequency directions, leading to a continuous decrease in transfer until reaching zero at 0 and $2\alpha$ frequencies^33,34^.

It is important to note however that a CTF is not always indicative of the actual strength of transfer, particularly at lower doses, and a noise-normalised transfer can be more appropriate^34,35^. The impact of noise on the CTF is especially noteworthy for iCoM and iDPC for which an ADF like CTF has been derived^2^, showing high low-spatial-frequency transfer. However, as the iDPC and iCoM signals involve dividing by the spatial frequency, they amplify the noise as well as the signal at low spatial frequencies, compared to the intrinsic 4D STEM transfer^2,36^. Indeed, the signal to noise ratio of iDPC peaks at around the frequency corresponding to the convergence semi-angle and steadily decays to zero at zero spatial frequency^2^.

Given that the same fundamental 4D STEM information transfer is utilised, similar considerations of noise should also apply to iterative ptychography methods generally. Also, for superresolution in iterative methods the strength of the high frequency components can be seen to drop off relatively quickly with angle outside the bright field disk. As the dose is reduced the amount of scattering to angles outside the BF disk reduces and can become very low indeed at low doses meaning the iterative reconstruction resolution limit can potentially decrease to $2\alpha$, similar to SSB. It is furthermore noteworthy that frequencies away from the maximum transferred frequency can be boosted for any in-silico method, including SSB as shown by O’Leary et al.^34^, although care should be taken as one may also boost the noise.

**Fig. 4:**
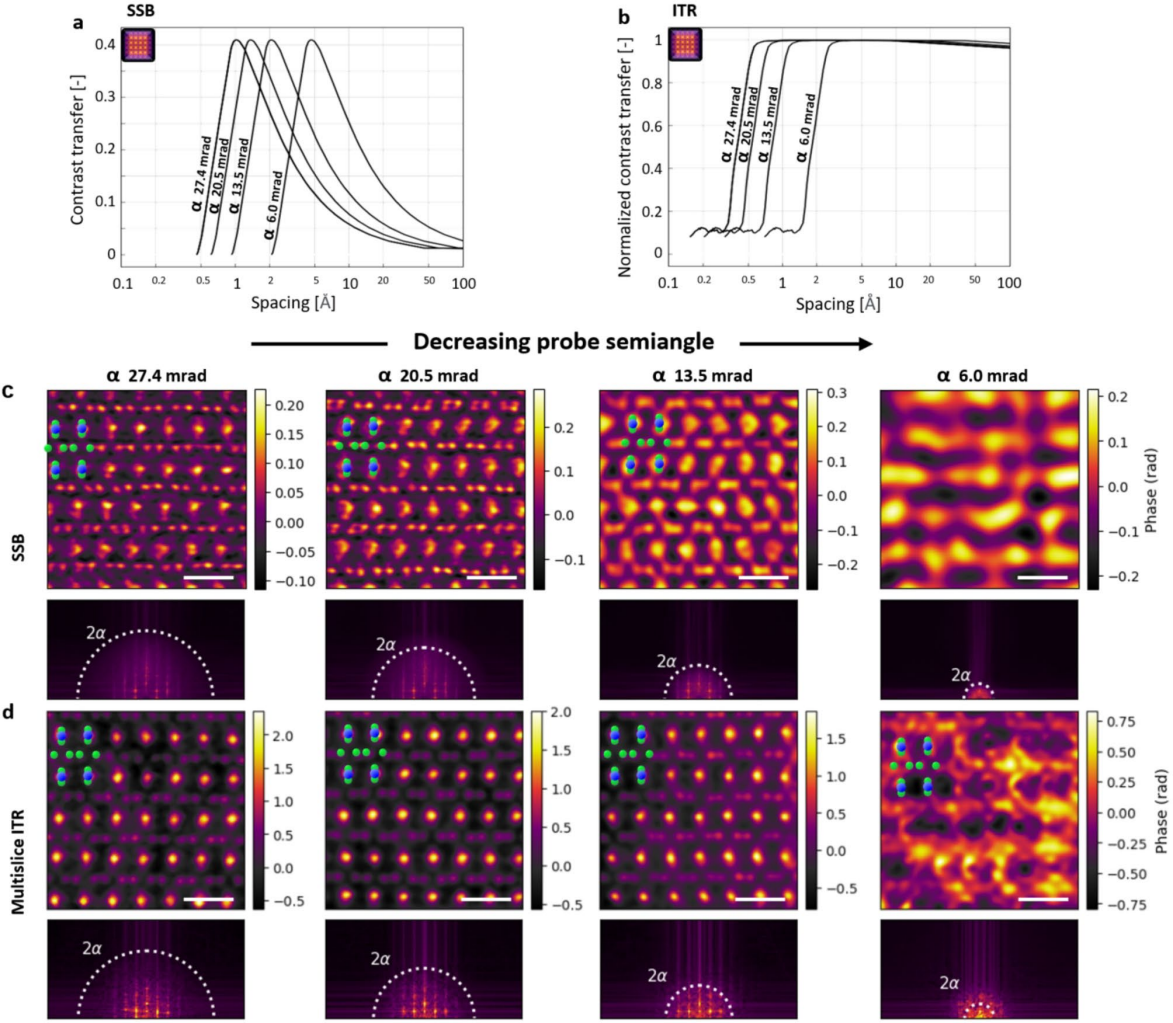
Sample driven electron probe semi-angle selection. (**a**) Phase CTF for SSB and (**b**) simulated phase CTF for ITR (simulated/reconstructed using abTEM) displayed as a function of real space spacing. Notice how the ITR image quality with probe semi-angle of 13.5 mrad benefits from non-zero CTF transfer beyond 2$\alpha$. SSB (**c**) and ITR (**d**) phase images together with their power spectra for various probe semi-angles. Dotted white circles show the 2$\alpha$ limit. Overlaid graphics demonstrates $\hbox {SmB}_{6}$ atomic structure (green filled circles B, blue filled circles Sm atoms respectively). Scale bars correspond to 5 Å.

*ptychoScopy* allows the x-axis of the CTF to be displayed in various units: $\omega$ (multiples of $\alpha$), miliradians, or real-space lattice distances Å corresponding to the respective scattering angles. The convergence semi-angle can then be adjusted to optimise the CTF for the range of frequencies of greatest interest in a sample. CTFs plotted in *ptychoScopy* are shown in Figure 4a,b for SSB and ITR respectively. Note that several probe angles can potentially be used consecutively to produce an ultra-wide CTF available for the reconstruction^37,38^, although multiple scans are required for this.

For demonstration, experimental datasets of the same field of view of the $\hbox {SmB}_{6}$ were acquired with a constant maximum captured scattering angle of 79.8 mrad and four different probe convergence semi-angles: 27.42 mrad (2.9$\alpha$), 20.52 mrad (3.8$\alpha$), 13.52 mrad (5.8$\alpha$), and 6.02 mrad (13.1$\alpha$). The structure of the sample is well transferred to the resulting phase images in the case of 27.42 and 20.52 mrad angles for both SSB (Fig. 4c) and ITR (Fig. 4d), using the maximum-likelihood based reconstruction engine^39,40^ within PtychoShelves^41^. Decreasing the convergence semi-angle to 13.52 mrad degrades the quality of the SSB image. It is due to the frequencies of interest, such as the B-B spacing of 1.2 Å, being closer to the resolution limit and are therefore less strongly transferred. This is not the case for ITR where superresolution extends the transfer significantly beyond the conventional 2$\alpha$ limit at this dose. SSB is therefore more sensitive to choosing too small of a convergence semi-angle compared to ITR for which the signal from all captured scattering angles is used during the reconstruction process, assuming sufficient signal is present.

Finally, the lowest angle, 6 mrad does not transfer sufficiently high frequencies for this material to be well resolved with either direct or iterative methods. With the maximum captured angle now corresponding 13.1$\alpha$, there are nearly no electrons detected at the high scattering angles needed for superresolution to overcome the small convergence semi-angle. Our results were obtained with a moderate electron dose of 136,000 e/Å$^{2}$.

### Detector array size and its impact on reciprocal space sampling

**Fig. 5:**
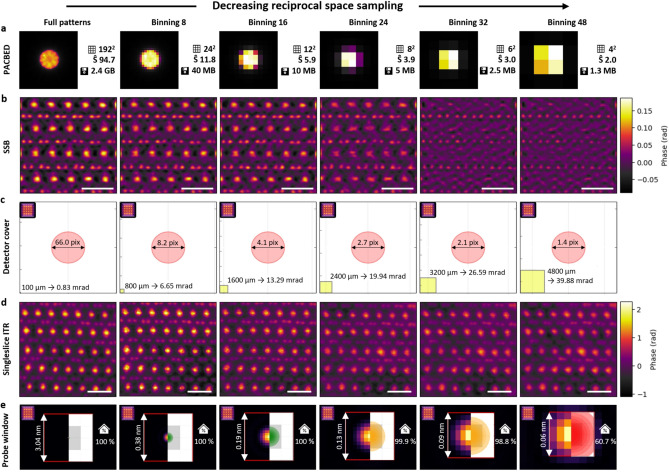
Reciprocal space sampling. (**a**) Position averaged convergent beam electron diffraction (PACBED) patterns at numerous binning levels. (**b**) Phase images from SSB reconstructions giving recognisable atomic features down to BF disk of $\hbox {4}^{2}$ pixels (binning 24). (**c**) Detector pixel size growth with increasing binning with respect to the BF disk (yellow squares). (**d**) ITR shows capability to work even with $\hbox {4}^{2}$ pixels in diffraction pattern (binning 48). (**e**) Simulated electron probe overlaid by probe window size (red square) and schematic drawing of the probe (right part). The fraction of probe intensity covered by the probe window is significantly reduced only at the highest binning of 48. Note that the probe window size decreases with increasing binning levels, inversely proportional to the effective pixel size. Bars correspond to 5 Å.

4D STEM datasets consisting of a full diffraction pattern for each scan position, are far larger than a conventional STEM dataset. Reducing the number of pixels per diffraction pattern can therefore provide faster processing and lower memory requirements, although, if the reduction is too severe, it can reduce the peak signal-to-noise ratio^42^. Additionally, many detectors can be accelerated using binning, in which their native pixel grid is reduced to a smaller effective number of pixels. To test the impact of the detector pixel dimensions on reconstruction quality, we used post-collection binning on a 27.42 mrad convergence semi-angle dataset we acquired with a detector coverage of 2.9$\alpha$. For our $\hbox {192}^{2}$ pixel detector this provides a balance of significant angles beyond $\alpha$ for superresolution while retaining detailed structure within the BF disk in the original unbinned dataset. Figure 5a displays the results of applying different amounts of post-acquisition binning together with applied sampling and the size of the dataset. We tested pixel dimensions down to $\hbox {4}^{2}$ pixels (48 binning).

For SSB reconstructions the minimum acceptable BF disk diameter was found to be four by four pixels in the data binned down to an 8$\times$8 grid (Fig. 5b, c). This corresponds to a binning factor of 24. A BF disk diameter of approximately two by two pixels, corresponding to a binning factor of 32, proved to be insufficient for generating a reliable reconstruction (Fig. 5b binning 32). Interestingly, in the latter case, while the low frequency reconstruction fails, the boron positions, corresponding to a high frequency transfer remains visible (Fig. 5b binning 32 and 48). This is likely due to the fact that at high binning most trotters, corresponding to low frequencies, overlap within the same two pixels which hinders information transfer. Only in case of highest frequencies, such as 1.9$\alpha$, there is a partial overlap with a neighbouring four pixels, as illustrated in Fig. S2.

In comparison, we observed the ability of ITR to reconstruct a fairly reasonable image even with a binning of 48, i.e. $\hbox {4}^{2}$ pixels for the full diffraction pattern. We used a singleslice ptychographic reconstruction, which is less sensitive to low reciprocal space sampling compared to multislice reconstructions as can be seen in Fig. S4. Although losing some image quality (Fig. S3a), the main sample structure is still preserved (Fig. 5d binning 48). The degradation of image quality is caused by reciprocal space undersampling, which occurs when the probe exceeds half of a probe window (Fig. 5e), i.e. probe sampling in frequency space falls below the Nyquist rate^43^. To avoid this, the probe diameter ($d_\mathrm {probe\_max}$) should not exceed half the size of the computational window of the probe (the probe window in short; $D_{\mathrm {\psi }}$) given by

1 $$\begin{aligned} d_\mathrm {probe\_max} = \frac{D_{\mathrm {\psi }}}{2} = \frac{\lambda }{2\Delta \theta } \approx \frac{\lambda L}{2 p_\textrm{eff}}, \end{aligned}$$

where $\lambda$ is the electron wavelength, $\Delta \theta$ is the angular range covered by a single detector pixel, *L* the distance from the sample to the detector plane (the effective camera length) and $p_\textrm{eff}$ is the effective physical detector sampling pitch (including binning). In this scenario, *ptychoScopy* will give a warning in the appropriate column of the camera length guide, as indicated in Figure 3m. However, it is important to note that ptychography allows for a trade-off between reciprocal and real-space sampling^28,44^, where relaxing the sampling requirements in reciprocal space is compensated by finer sampling in real space.

### Optimal real space sampling and its influence on reconstruction quality

**Fig. 6:**
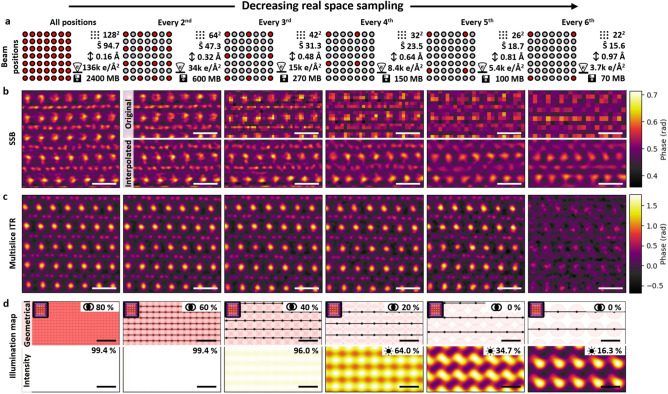
Real space sampling. (**a**) Schematic drawing of the beam positions: red filled circles used, gray filled circles skipped. (**b**) SSB reconstructed phase images from the reduced number of beam positions and interpolated to original number of $\hbox {128}^{2}$ real-space pixels. (**c**) ITR phase images giving interpretable results up to every $\hbox {5}^{th}$ beam position. (**d**) Illumination maps in form of geometrical overlap generated by *ptychoScopy*, where individual probe positions are represented by light red circles and intensity map based on experimentally determined probe. Bars in (**b**, **c**) correspond to 5 Å, in (**d**) to 1 Å.

To examine the reconstruction quality as a function of real-space sampling density, the original dataset shown in Fig. 6 has been modified by periodically skipping a fraction of the beam positions (taking every $\hbox {2}^{nd}$ to every $\hbox {6}^{th}$ position). As a result, the real-space sampling step in these datasets was increased from its original 0.16 Å up to 0.97 Å as shown in Fig. 6a. With a constant field of view, both the size of the resulting datasets and the cumulative electron dose are reduced by a factor of $(\textrm{skip}+1)^2$.

As the pixel size in SSB reconstructions is determined by the scanning step size ($\Delta R = Pix_{\textrm{SSB}}$), the maximum appropriate step size can be derived from the CTF $2\alpha$ limit, which is determined by the probe semi-angle. The lateral resolution ($r_{x,y}$)^30,45^ then can be calculated as

2 $$\begin{aligned} r_{x,y} = \frac{\lambda }{\sin {(2\alpha )}}. \end{aligned}$$

Considering the Nyquist–Shannon sampling theorem for minimally sampling this spacing, this leads to the following formula:

3 $$\begin{aligned} \Delta R_{\textrm{SSB}} = \frac{\lambda }{2\sin {(2\alpha )}} \approx \frac{\lambda }{4\alpha }, \end{aligned}$$

using the small-angle approximation. For the used probe semi-angle of 27.4 mrad the recommended step size corresponds to 0.23 Å. However, the sampling can be reduced to larger step sizes up to the level of the sample periodicity, which has to be appropriately sampled, i.e. to resolve the 1.2 Å B-B atomic distance, a Nyquist sampling rate of 0.6 Å  is necessary (Figure 6b every $\hbox {3}^{rd}$). Additionally, various reconstruction results from real vs. reciprocal space sampling combinations can be found in Fig. S5. To enhance the visual quality of the reconstructed image consisting of fewer pixels with increasing skipping, we have applied Lanczos interpolation (part of the Matplotlib Python package) to upsample the image back to the original $\hbox {128}^{2}$ pixels (see Figure 6b and Figure S5). This mitigates the pixelation present in the undersampled image and makes the boron atoms stand out.

For ITR, the situation is more complex as the scanning step size is decoupled from the reconstruction pixel size ($\Delta R \ne Pix_{\textrm{ITR}}$). The reconstructed pixel size is determined by the maximum collected scattering angle,

4 $$\begin{aligned} Pix_{\textrm{ITR}} = \frac{\lambda }{\theta } \approx \frac{\lambda L}{Mp}, \end{aligned}$$

where $\theta$ is the maximum detected angle on the detector, *M* the half width of the detector array in pixels and *p* the physical size of the pixels^46^. The pixel size in ITR can be reduced in post-acquisition by padding the diffraction patterns with zeros (*Padding* in Fig. 2b) to artificially increase the maximum detector coverage angle^12,16,47^.

The real-space sampling rate (*SR*) can be defined in various ways with respect to the diameter of the beam. Outside the common approach of geometric overlap of two adjacent beam positions, shown as $SR_{overlap}$ below, the linear/areal (over)sampling^18^ can be defined as

5 $$\begin{aligned} SR_{\textrm{overlap}} = \frac{d_{\textrm{probe}}-\Delta R}{d_{\textrm{probe}}}\cdot 100 \ [\%], \hspace{0.5cm} SR_{\textrm{linear}} = \frac{d_{\textrm{probe}}}{\Delta R}, \hspace{0.5cm} SR_{\textrm{areal}} = \frac{\pi (d_{\textrm{probe}}/2)^{2}}{\Delta R^{2}} \end{aligned}$$

where the $d_{\textrm{probe}}$ is the electron probe diameter (see Figure 6d). As all three methods describe the same feature, only the geometric overlap is shown in the present manuscript, but all are included in *ptychoScopy*. Notably, the simple geometrical overlap model is not always sufficient to describe the beam overlap, since even with zero geometrical overlap successful reconstructions are still possible as illustrated using a step size of 0.81 Å in Fig. 6c,d and Fig. S3b.

It has been suggested that achieving an ideal probe overlap requires constant illumination across the sample leading to a uniform dose distribution^28^. To analyse this experimentally, an illumination map was created by placing the intensities of an ITR estimated probe in scanning grid positions (Fig. 6d) and consequently, the uniformity of the illumination *U* has been introduced and defined as shown in Figure S6.

With the small probe diameter of $\sim$0.8 Å, used in this study, even with side lobes providing extra overlap, there is a strict limit for real space sampling (occurring between sampling every $\hbox {5}^{th}$ and $\hbox {6}^{th}$ probe position in Fig 6c). The aforementioned case of Fig. 6c, in which every $\hbox {5}^{th}$ beam position resulted in a reasonable reconstruction despite having zero geometrical overlap, can be subsequently explained by the uniformity of intensity illumination, which was found to be 34.7 %.

Moreover, for proper reconstructions, the following sampling criterion has been shown to hold^28,48^

6 $$\begin{aligned} \Delta \theta \Delta R \le \frac{\lambda }{2}. \end{aligned}$$

The amount of sampling $(\widehat{S}_{x,y})$ can then be defined as

7 $$\begin{aligned} \widehat{S}_{x,y} = \frac{\lambda }{2\Delta R \Delta \theta } \approx \frac{\lambda L}{2\Delta R p_\textrm{eff}}, \end{aligned}$$

with the criterion that has to be fulfilled in both *x* and *y* directions becoming $\widehat{S}_{x,y}\ge 1$^44,48^. In *ptychoScopy* we assume a homogeneous scanning grid with $\Delta R_{x}=\Delta R_{y}$, although it should be noted that some reconstruction software can handle any scan pattern, e.g. PtychoShelves^41^ or py4DSTEM^49^.

This sampling equation for ITR ptychography establishes a relationship between real and reciprocal space sampling allowing the use of tiny detection arrays with few pixels only for cases where dense real space sampling is used, as shown by Zhang et al.^50^. On the other hand, there will be limitations in the case of larger probes (defocused probe) and sparse real-space sampling. Therefore, the combination of small individual working real and reciprocal space sampling such as that shown in Fig. S8 step 0.81 Å array $6^{2}$ pix, does not work since the sampling requirement is not satisfied - i.e. $\widehat{S} = 0.6$.

### Constraints for defocused probe ptychography

Increasing the size of the defocused electron beam enables one to cover the sample with fewer probe positions allowing a significant reduction in the applied electron dose for a given probe current and data collection rate. Considering the relation between the probe size and the computationally reconstructed probe window mentioned above, see Eq. (1), the maximum recommended defocus ($\Delta f_\textrm{max}$) can be calculated as

8 $$\begin{aligned} \Delta f_\textrm{max} = \frac{d_\mathrm {probe\_max}}{2\tan (\alpha )} \approx \frac{d_\mathrm {probe\_max}}{2\alpha }. \end{aligned}$$

**Fig. 7:**
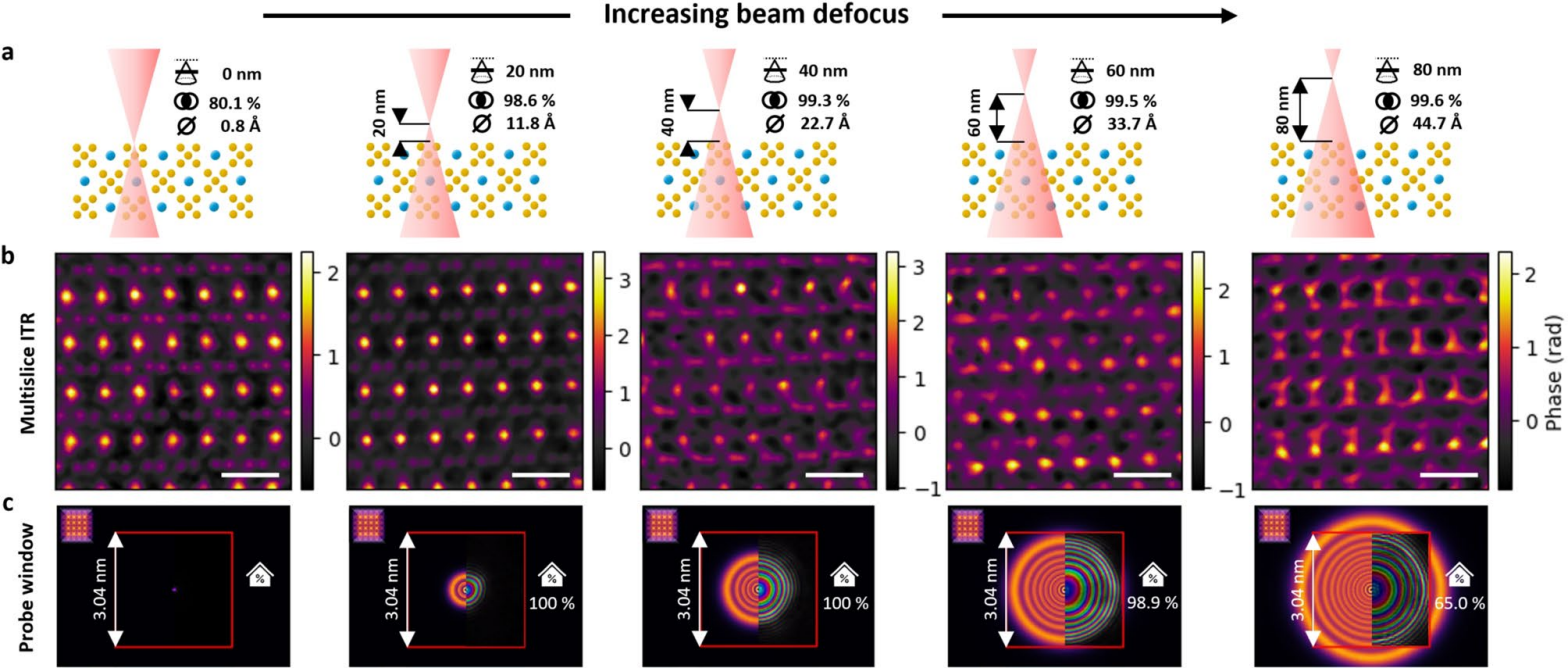
Reconstruction with defocused probe. (**a**) Schematic drawing of the beam defoci. (**b**) ITR phase images. (**c**) Simulated electron probe using abTEM^32^ (left half) complemented by its reconstruction using PtychoShelves^41^ (right half). Fraction of probe intensity covered by the probe window is decreasing with increasing beam defocus. The probe window is highlighted by red square. Scale bars correspond to 5 Å.

To demonstrate the influence of an exceedingly large electron probe for a fixed probe window, datasets have been acquired with increasing defocus, i.e. from 0 up to 80 nm while keeping all other parameters constant (Fig. 7a). As shown in Fig. 7b, the datasets taken with 60 nm and 80 nm defocus drop significantly in quality compared to those acquired with zero defocus, i.e. focused, or 20 nm defocus or even 40 nm defocus. The drop in quality is caused by undersampling the diffraction pattern (Fig. 7c) leading to aliasing artifacts within the reconstructed images. This can be suppressed with a low-pass filter at the cost of high frequency loss or by creating a sufficiently large probe window.

In *ptychoScopy* the probe window and related maximum beam defocus (Eq. 8) is based on the applied sampling $\widehat{S}_{x,y}$ and shown in the camera-length-guide (Fig. 3m).

### Low dose imaging and resolution drop

**Fig. 8:**
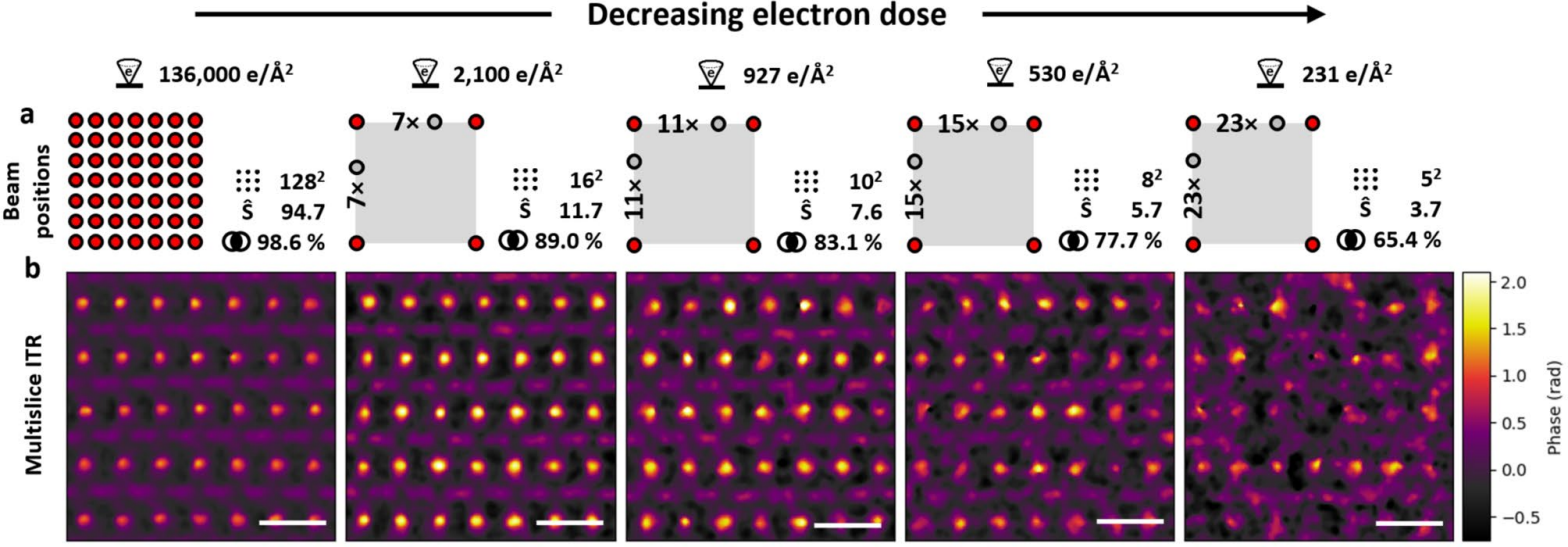
Low dose reconstruction. (**a**) Schematic drawing of the beam positions: red filled circles used, gray filled circles skipped. (**b**) ITR phase images of the same area reconstructed with various electron doses (based on dataset with 20 nm defocus). The results show significant reduction in sample structure visibility with decreasing electron dose, where Boron atom columns can be still recognised down to dose of 530 e/Å$^{2}$ but at 231 e/Å$^{2}$ it is very difficult to distinguish both, Samarium and Boron atoms from background. Bars correspond to 5 Å.

To examine the effect of the cumulative dose on the quality of the ptychographic reconstruction, as well as to decouple the influence of cumulative electron dose from beam overlap, an oversampled, defocused dataset was acquired and subsequently used with skipped beam position during the post-processing step (Fig. 8). Within the 20 nm defocus dataset, from 7 to 23 beam positions have been skipped in the reconstructions to reduce the utilised electron dose from 136,000 down to 231 e/Å$^{2}$ (Fig. 8a). As can be seen in Fig. 8b, the quality of the reconstruction drops very significantly with electron doses lower than 530 e/Å$^{2}$, even with a sufficient geometrical beam overlap of 64.4 % for the dose of 231 e/Å$^{2}$.

Dose reduction can be necessary based on specific sample requirements and can compromise the reconstruction image quality. In addition, with the reduction of applied dose, it becomes extremely crucial to optimise the reconstruction settings, in dose range 100 e/Å$^{2}$ to 50,000 e/Å$^{2}$ as shown by Cao et al.^19^. On the other hand, high-dose reconstructions are less sensitive to quality degradation.

### Multislice reconstruction limitations

The depth resolution in multislice ITR, $r_{z}$, can exceed the conventional aperture limited depth resolution defined by the convergence semi-angle of the beam^51^, and depends on the maximum detected angle, scattering power of the specimen, and the dose^11^. In x-ray ptychography the depth resolution is given by^30,45^

9 $$\begin{aligned} r_{z}= \frac{\lambda }{2\sin ^{2}{(\theta /2)}}, \end{aligned}$$

where $\theta$ represents the maximum scattering angle captured on the detector. While this formulation does not explicitly include a dose, the depth resolution of multislice electron ptychography appears to approach this limit as dose is increased^11^. We therefore believe this represents a reasonable estimate for a lower bound for the minimum useful slice thickness in multislice electron ptychography. As lower doses decrease the depth resolution, it would seem reasonable to use thicker slices at lower doses. One could potentially account for this by using a smaller effective maximum scattering angle. In *ptychoScopy* the information about minimal slice thickness can be found in the camera-length-guide (Fig. 3m).

## Discussion

*PtychoScopy* was developed to optimize experimental parameters in electron ptychography with an emphasize on balancing the dose efficiency, resolution and imaging parameters across varying sample conditions. The tool offers flexibility in defining microscope specific optical parameters and precision with respect to selection of parameters, such as probe size, defocus, sampling in real and reciprocal space, while being correlated with the desired reconstruction strategy.

Careful selection of probe semi-angle showed its critical influence on achievable resolution and contrast, where direct reconstructions with SSB method was, as expected, subject to resolution degradation when selecting smaller semi-angles (13.5 mrad) as compared to ITR methods, since the later utilizes superresolution using the signal from the full diffraction patterns. While achieving resolution as high as 0.44 Å with a smaller convergence semi-angle of 7.5 mrad with probe defining angles up to 7.6$\alpha$ is possible as previously shown by Nguyen et al.^52^ the total dose used in that study was 420,000 e/Å$^{2}$, which underlines the importance of high angle scattering and sufficient electron dose for achieving superresolution. Therefore, the importance of matching probe semi-angles to desired sample frequency using corresponding contrast transfer function as a guide has been reaffirmed and included in *ptychoScopy*.

To overcome large data volumes and the consequently more computationally demanding data handling from ptychographic reconstructions, the minimal detector array size sufficient for reasonable reconstructions has been investigated. While SSB exhibits limitations below a BF disk diameter of 4 pixels, ITR maintained interpretable reconstruction results and quality down to a 4$\times$4 pixel array (48$\times$48 pixel binning), which opens the possibility to considerably reduce the dataset size (shown from 2.4 GB down to 1.3 MB), accelerating processing to even on-the-fly image reconstructions using the later method. Consequently, depending on the desired outcome, it is possible to reduce the dwell time of a hybrid pixel detector when applicable by binning.

Dose rate optimisation as well as the total amount of dose, i.e. cumulative dose, is often the main limitation for electron microscopy of radiation sensitive samples. Therefore reducing and controlling the electron dose is often of high interest. This can be achieved by reducing the pixel dwell time, real space sampling rate or lowering the probe current. While reducing the dwell time can in some cases be achieved by pixel binning, in this study a Dectris Arina detector has been used. This offered dwell time reduction down to eight microseconds, corresponding to 120kHz readout frequency, and is the fastest frame-based detector currently available in the market. An alternative can be introducing fast beam blanking to reduce the total exposure^53^ or using an event driven detector, such as the Timepix3, which already allows sub microsecond dwell times, with a requirement of a limited probe current especially for a single chip setup, see Jannis et al.^54^.

In this study the extent of dose reduction and its interplay with real-space sampling has been investigated. It was achieved by skipping beam positions as a post-processing step, which can be mimicked experimentally by reducing the number of beam positions for a given scan area. While in SSB-based reconstructions, the upper limit of the step size is equivalent to the scanning step size being constrained by the Nyquist-Shannon sampling theorem, in the case of ITR methods the upper limit of image resolution is given by the maximum collected scattering angle.

While defocusing the probe allowed further dose reduction via increased step size, the trade-off came with constrains related to the probe window given by reciprocal space sampling. Very large probes result in the undersampling of fine structure in diffraction patterns as each pixel integrates a significant area of the scattered intensity. With sufficient combined sampling, synthetic detector data can be created to fulfil the probe window size limitation^48^. In this study, the dose threshold below which the resolution of the resulting reconstruction has been diminished was identified to be at 530 e/Å$^{2}$.

As shown in this work, proper data collection design plays a crucial role in enabling optimal ptychographic experiments, reconstructions and image interpretability. With experimental design optimisation, datasets can be collected at higher speed, saved with lower disk space, and processed faster. Moreover, the optimal real-space sampling, dwell time, and defocus allows reducing the applied electron dose, mitigating sample damage and sample drift which can be a limiting factor in high-resolution imaging. Therefore, by leveraging the tools like *ptychoScopy*, researchers can unlock the full potential of this high-resolution imaging technique.

## Methods

### Figure legends

A set of symbols has been introduced to simplify the figures presented. $D_{\mathrm {\psi }}$ is the probe window size and $d_{\textrm{probe}}$ the probe diameter.

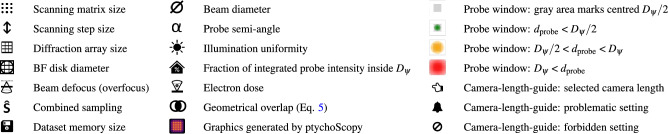



### Sample preparation

For the acquired datasets, a thin lamella of an $\hbox {SmB}_{6}$ crystal was prepared by FIB/SEM. The sample was chosen as an electron dose tolerant crystal consisting of heavy ($_{62}^{150}\text {Sm}$) and light ($_{5}^{10}\text {B}$) elements. The crystal was oriented along the [110] zone axis in order to be able to image atomic columns of only light (B) or only heavy (Sm) species, respectively. The distance between B-B columns is 1.21 Å  and 1.71 Å, that of Sm-Sm columns is 2.92 Å ad 4.13 Å, respectively. The atomic arrangement can be found in the Fig. S7.

### Electron microscopy

All data were acquired on a JEOL ARM200F (NeoARM) TEM/STEM equipped with a cold FEG and a probe corrector with a nominal resolution of 0.78 Å in STEM mode and 2.3 Å in TEM mode. The datasets were acquired with an ARINA detector (Dectris) with a framerate of 20 kHz with full detector read out ($\hbox {192}^{2}$ pixels) and 100 kHz with binning 2. Datasets were acquired with the following parameters: magnification 3$\cdot 10^{7}$, scanning matrix $\hbox {512}^{2}$ pixels, probe aperture 40 $\upmu$m (probe semi-angle of 27.42 mrad), probe current 9C (11 pA), nominal camera length 8 cm (corresponding to an effective camera length of 12.12 cm), a total detector cover angle of 79.8 mrad (respectively 2.91$\alpha$ for the 40 $\upmu$m aperture) and a dwell time of 50 $\upmu$s without binning (diffraction pattern size $\hbox {192}^{2}$ pixels). When needed, several variations of a chosen parameter were taken (apertures 30 $\upmu$m: 20.5 mrad, 20 $\upmu$m: 13.5 mrad, 10 $\upmu$m: 6.0 mrad) and beam defocus (0 nm, 20 nm, 40 nm, 60 nm, 80 nm).

The focused probe diameter of 0.8 Å  used in this study is defined by microscope manufacturer JEOL as the area including 59 % of the total beam intensity.

**Scanning step calibration**: The scanning step size calibration for a given magnification and field of view was based on the known inter-atomic distances of the $\hbox {SmB}_{6}$ crystal in [110] orientation, where the Sm to Sm distances are 2.92 and 4.13 Å respectively. The line of 23 Sm atoms was imaged, the distance in pixels was counted, and the correction factor between real and nominal pixel size was calculated.

**Probe semi-angle and camera length calibrations**: Probe convergence semi-angle was determined using Silicon crystal of a known orientation. Based on the known Si-reflections and the corresponding Bragg angle, the semi-angle is proportional to the ratio between the spacing of Bragg discs and the corresponding aperture diameter. Camera length calibrations at 200 kV acceleration voltage, were derived from [220], and [002] spot distances of the Silicon crystal based on camera equation ($R_{hkl} \cdot d_{hkl}=\lambda \cdot L$) and Bragg’s equation with small angle approximation.

**Pumping aperture angle restriction (PAAR)**: With calibrated single pixel angular coverage, the camera length can be lowered until the differential pumping aperture between the TEM column and the camera chamber becomes visible on the pixelated detector. Based on the calibrated angular range of the bright field disk, the maximum angle passing through the aperture has been determined.

### PtychoScopy

In-depth documentation and pipelines together with examples and the code itself can be found in the Gitea repository of https://gitea.psi.ch/EMF/ptychoscopy.gitptychoScopy tool. To ensure proper functionality, we recommend setting up a specific Python environment with tested versions of the required packages. The *ptychoscopy.yml* file for building the same environment as used in this study is included in the repository.

### CTF calculation for ITR

We followed the same steps as used by O’Leary et al.^34^: 1. Simulation of isolated atom potential. 2. Simulation of 4D STEM data based on the potential (no Poisson noise added). 3. Ptychographic reconstruction. 4. Deconvolution of the simulated atom potential from the ptychographic phase, i.e. division in Fourier space. 5. Calculation of azimuthal average. Both simulation and ptychographic reconstruction were performed with abTEM^32^ package using ePIE method for reconstruction.

### Data pre-processing and ptychographic reconstruction

The acquired diffraction patterns were corrected for both flat-field and dead pixels. Except for “low-dose imaging”, one-sixteenth of the taken dataset ($\hbox {512}^{2}$ beam positions) was used for the presented reconstructions ($\hbox {128}^{2}$ beam positions) - the field of view was reduced from 8 nm $\times$ 8 nm to 2 nm $\times$ 2 nm (various skipping or/and binning was applied). The “low-dose imaging” (Figure 8) data characterised by high skipping (from 7 to 23 beam positions) were created from the entire dataset of $\hbox {512}^{2}$ and the final reconstructed image was cropped to a field of view of 2 nm $\times$ 2 nm.

All reconstructed phase images acquired from different experiments contain their own corresponding color bars. For the images generated from the same experiment and subsequently postprocessed into different binning or skipping variants, only one shared color bar has been used.

**Single side band ptychography reconstructions** were performed with the open source https://gitea.psi.ch/EMF/ptychoscopy.gitpyPtychoSTEM available on Gitea. The code was locally modified at PSI to read the Dectris ARINA data format in the form of *.h5* files. The reconstruction was set as follows: probe_angle = 27.42 mrad (for 40 $\upmu$m aperture), step_size = 0.15903 Å (without skipping), voltage = 200 kV, rotation = $\hbox {115.1}^\circ$ (angle between scan and detector), threshold = 0.4 (for BF disk localisation). **Iterative ptychographic reconstructions** were performed in https://www.psi.ch/en/sls/csaxs/software PtychoShelves[41]. For both single- and multislice reconstruction the MLc engine[40] was used. The main reconstruction parameters are shown in Table S2. In the case of multislice reconstructions, the top and bottom slices were not used for the final visualisation and the composite image was created from 7 out of 9 and 3 out of 5 slices.

## Supplementary Information

Supplementary Information.

## Data Availability

The datasets used in this study will be available upon request from the corresponding author. PtychoScopy is available to download from the Gitea repository: https://gitea.psi.ch/EMF/ptychoscopy.git.

## Glossary

**Focused probe:**: Within the manuscript, the beam has been always focused, but the position of the focus was adjusted with respect to the sample plane - i.e. a focused probe refers to a probe focused to a position at or near the entrance surface of the sample and a defocused probe refers to focusing the probe a significant distance away from the sample. In this paper, defocus was reached by overfocusing the corresponding objective lens. Note however that underfocus could also be used to focus the beam beyond the exit surface for defocused probe
**Probe window:**: The area used for probe characterisation during iterative reconstruction. Its size is equal to that of the detector array multiplied by the reconstructed pixel size. For proper sampling in Fourier space, the probe can only cover half of this size in real space.
**Superresolution:**: Within the manuscript, the term “superresolution” is used as higher information limit than conventional $2\alpha$ where $\alpha$ is probe semi-angle.
**Trotters:**: Batch of masks representing positions of double-overlapped diffraction disks for the whole range of spatial frequencies. They have the shape of two circles’ overlay - one on the BF disk i.e. unscattered beam and second changing its position (rotation around the centre and distance) i.e. scattered beam.
