# Supplementary material for: Ptychoscopy: a user friendly experimental design tool for ptychography
Source: Sci Rep. 2025 Jul 10;15:24959. doi: 10.1038/s41598-025-09871-6 (PMC12246451; doi:10.1038/s41598-025-09871-6)
Supplement: Supplementary file 1 — Supplementary Information. [file 41598_2025_9871_MOESM1_ESM.pdf]

Supplementary Information

Ptychoscopy: A User Friendly Experimental Design Tool for Ptychography

Radim Skoupy[1], Elisabeth Müller[1], Timothy J. Pennycook[2], Manuel Guizar-Sicairos[3,4], Emiliana Fabbri[5], and Emiliya Poghosyan[1,*]

[1] *PSI Center for Life Sciences, Paul Scherrer Institute, Villigen PSI, 5232, Swtizerland*
[2] *EMAT, Department of Physics, University of Antwerp, Groenenborgerlaan 171, Antwerp, 2020, Belgium*
[3] *PSI Center for Photon Science, Paul Scherrer Institute, Villigen PSI, 5232, Switzerland*
[4] *École Polytechnique Fédérale de Lausanne, Lausanne, 1015, Switzerland*
[5] *PSI Center for Energy and Environmental Sciences, Paul Scherrer Institute, Villigen PSI, 5232, Switzerland*
[*] *emiliya.poghosyan@psi.ch*

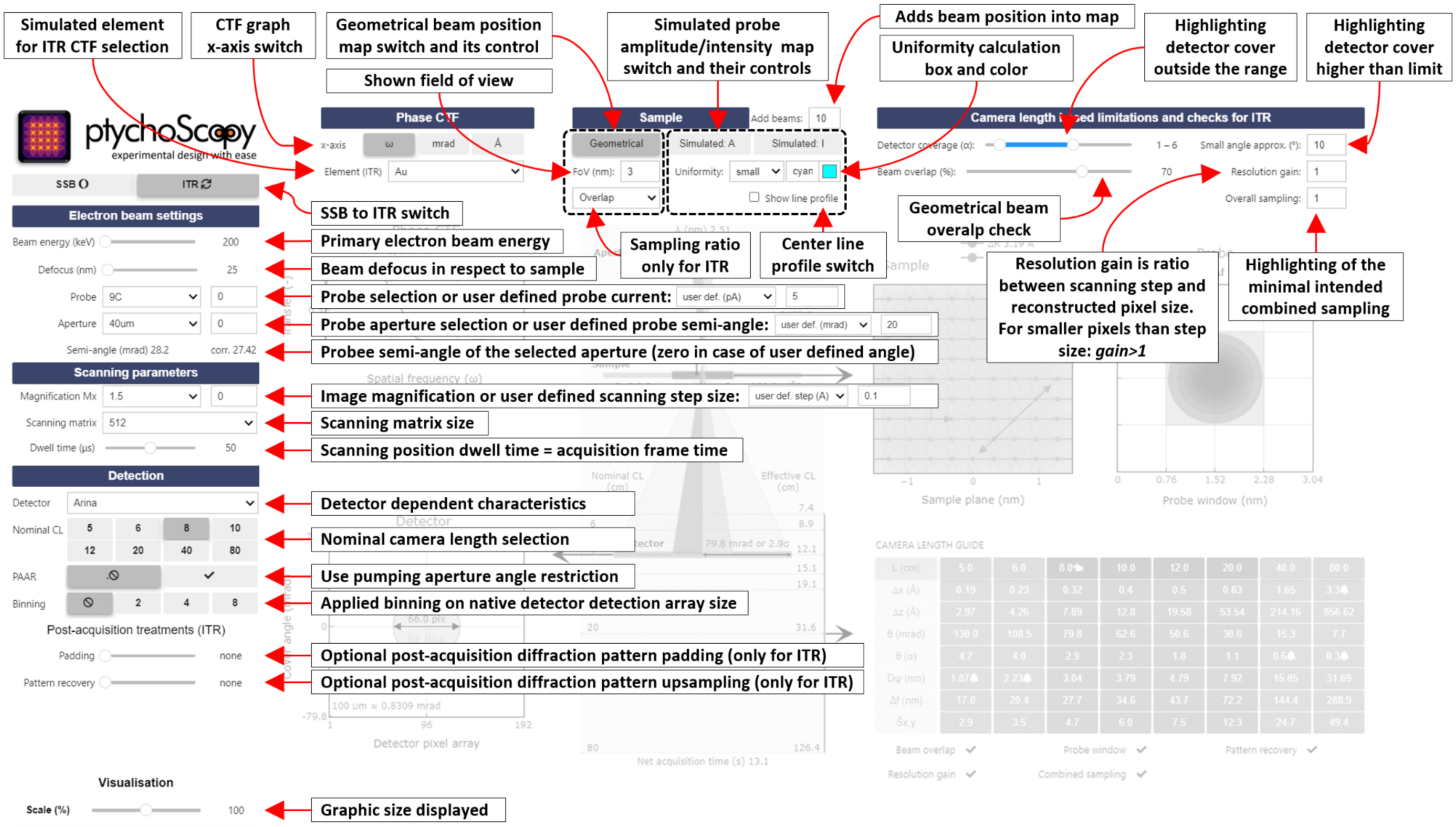

Figure S1. *PtychoScopy* controls description.

Table S1. Ptychography reconstruction algorithms.

<table>
<tr><td rowspan="9">Reconstruction methods[a]</td><td colspan="2" rowspan="2">Direct</td><td>SSB</td><td>Single Side Band</td><td>[20–24]</td></tr>
<tr><td>WDD</td><td>Wigner Distribution Deconvolution</td><td>[22–24]</td></tr>
<tr><td rowspan="7">Iterative</td><td>Sequential[b]</td><td>PIE</td><td>Ptychography Iterative Engine</td><td>[55–57]</td></tr>
<tr><td>Hybrid[c]</td><td>WASP</td><td>Weighted Average of Sequential Projections</td><td>[58]</td></tr>
<tr><td rowspan="5">Batch[d]</td><td>DM</td><td>Difference Map</td><td>[59]</td></tr>
<tr><td>CG</td><td>Conjugate Gradient</td><td>[5]</td></tr>
<tr><td>RAAR</td><td>Relaxed Averaged Alternating Reflections</td><td>[60]</td></tr>
<tr><td>ML</td><td>Maximum Likelihood</td><td>[39,61]</td></tr>
<tr><td>LSQ-ML</td><td>Least-Squares solver in generalised Maximum-Likelihood ptychography</td><td>[40]</td></tr>
</table>

[a] A large variety of methods exist and a detailed survey can be found in the chapter by Rodenburg and Maiden[57].
[b] Sequential projection algorithms treats each pattern sequentially.
[c] Hybrid approaches have been implemented such as the weighted average of sequential projections.
[d] Batch algorithms optimise the estimate of the exit wave using an entire batch of diffraction patterns.

Table S2. Main ITR parameters, where *Crop* means using only the middle part of each diffraction pattern - reduction from 2.9α to 1.45α, the *Modes* the number of incoherent probe modes and the *Position* the starting iteration of probe position refinement. When multiple lines can be found for one image reconstruction, a sequence of reconstruction steps was used where the output of the previous step was used as an initial guess for the corresponding one.

Figure	Slices	Crop	Modes	Iterations	Position
5d	single	No	9	500	100
4b, 6c, 7b	single	No	9	200	100
	multi: 9×2 nm	No	9	300	1
8b	single	Yes	4	300	100
	single	No	4	200	1
	multi: 5×4 nm	No	4	20	1

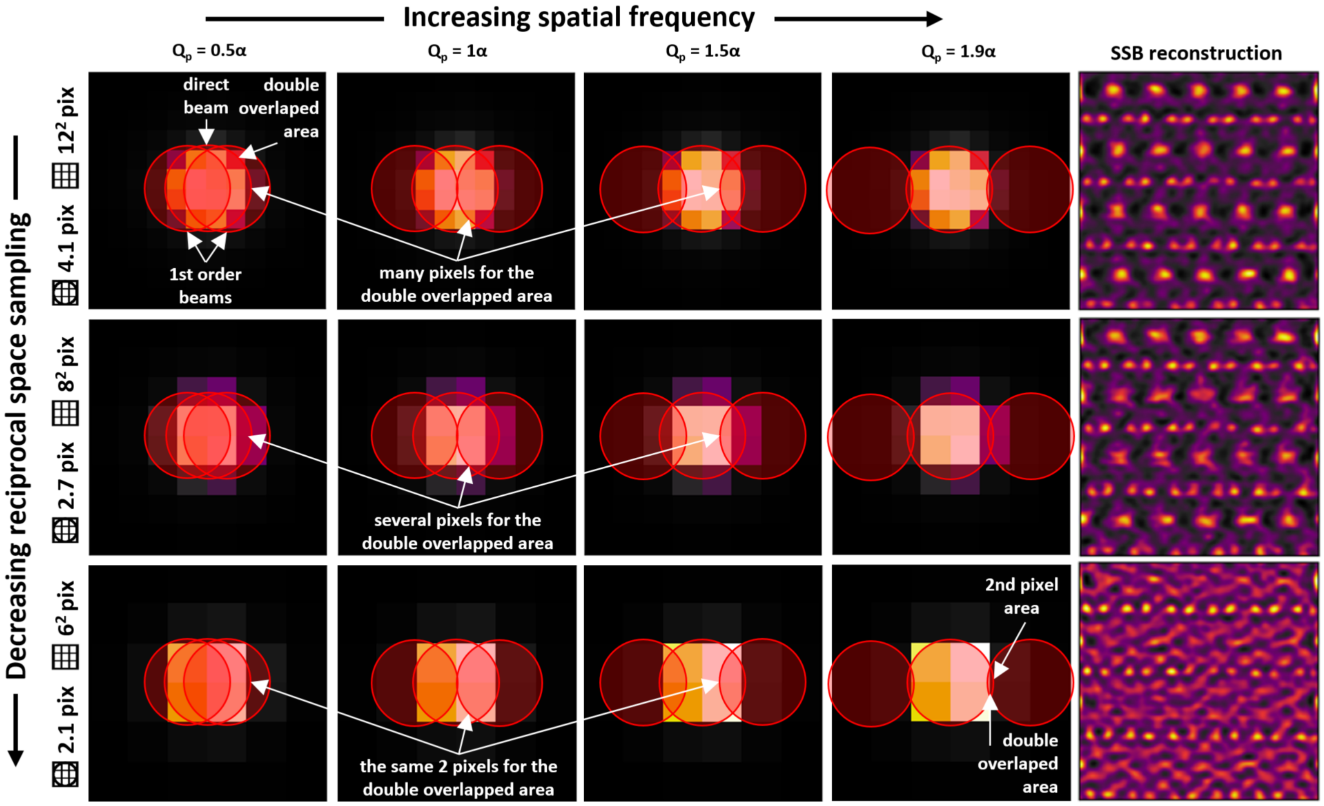

Figure S2. **Low reciprocal space sampling and SSB.** In case of very high binning (very small diffraction arrays), trotters of nearly all spatial frequencies (Q_p) are located over the same pixel resulting in close to no information transfer. However, in case of the highest spatial frequencies corresponding to 1.9α (4th column) and where the trotter cover very small part of second pixel from the centre, there is a high-resolution information transfer. As shown in this work, the bottom limit for the SSB method is BF disk diameter around 3 pixels. The real space of the specimen covers an area of 1.66 nm$\times$1.66 nm.

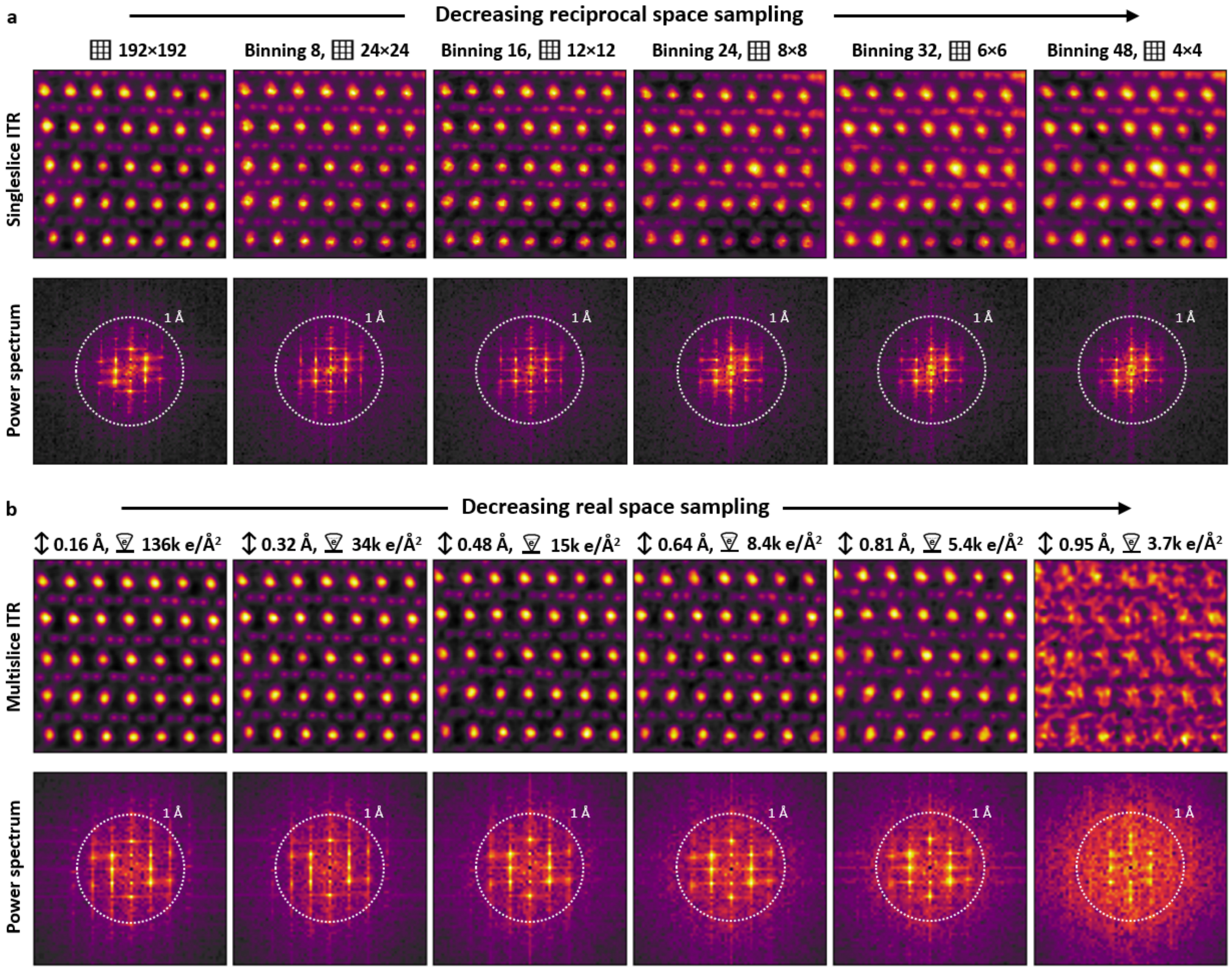

Figure S3. **Sampling related resolution loss and ITR.** The real space of the specimen covers an area of 2 nm×2 nm. (a) Decreasing reciprocal space sampling has only small impact to image resolution since the real space is densely sampled and the combined sampling $\widehat{S}$ is still sufficient. (b) In comparison, decreasing real space sampling, resulting in dose reduction, leads to inferior reconstruction quality, despite of the same maximum detected angle in reciprocal space. This is especially visible in the last reconstruction where apart from reduced total dose, the illumination uniformity is significantly reduced.

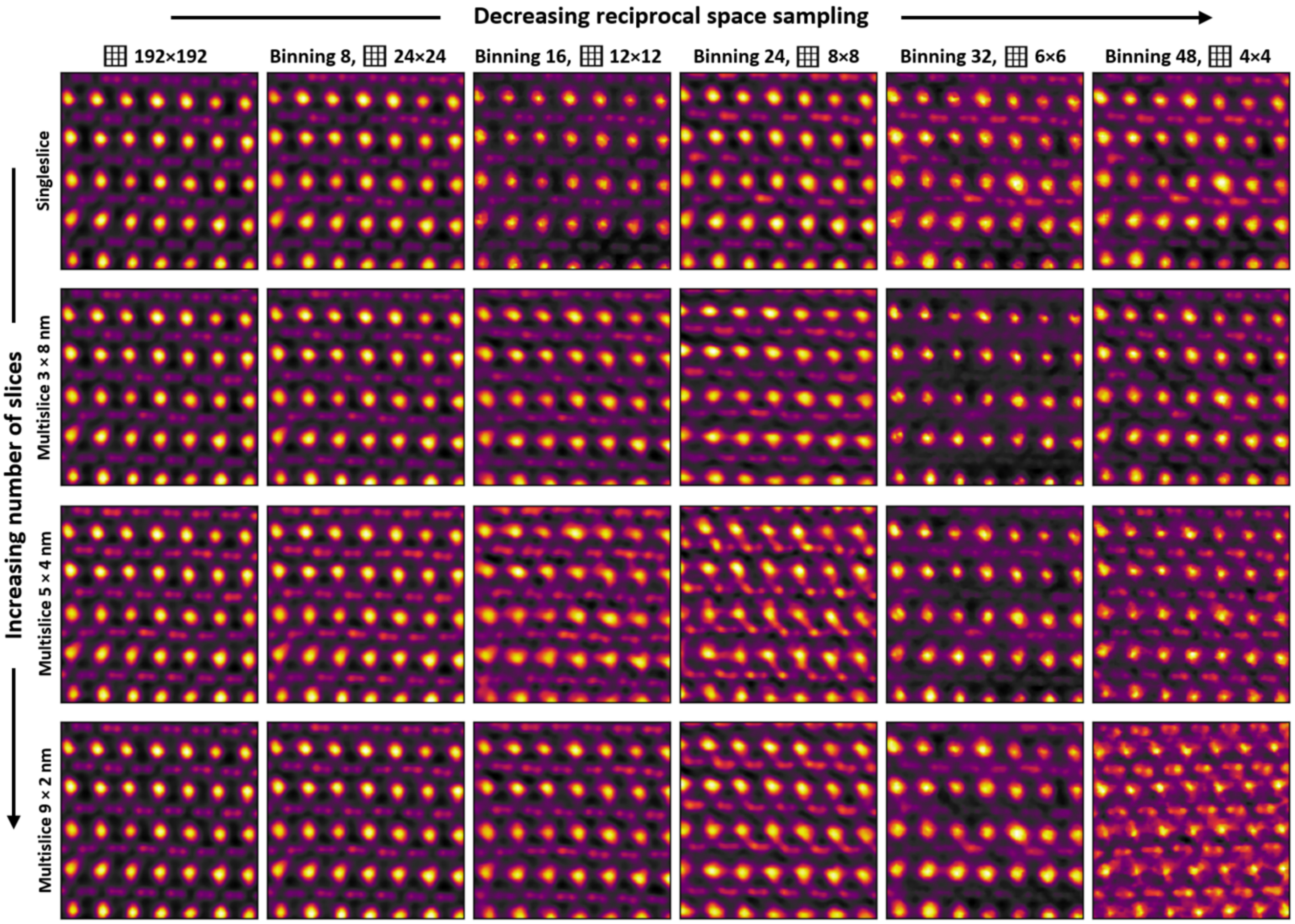

Figure S4. **Reciprocal space sampling and number of slices in ITR.** With lower reciprocal space sampling (higher binning) the reconstruction quality drops as visible in bottom right corner. The real space of the specimen covers an area of 2 nm×2 nm. The middle reconstructed slice is shown.

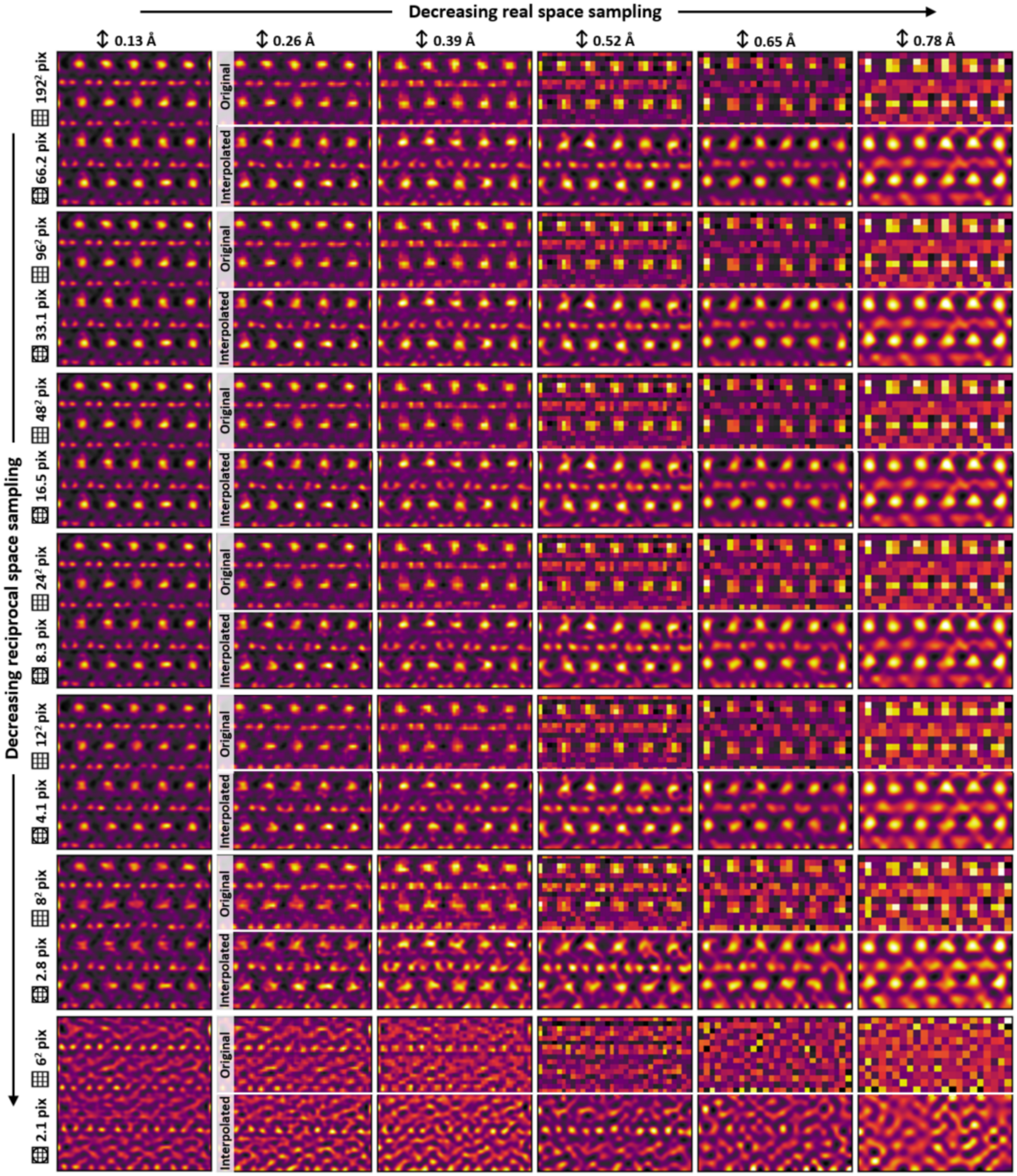

Figure S5. Combined real- and reciprocal-sampling reduction and its influence to reconstruction quality for SSB. The real space of the specimen covers an area of 1.66 nm×1.66 nm. Smaller step size is due to acquisition via different scanning unit compared to other datasets.

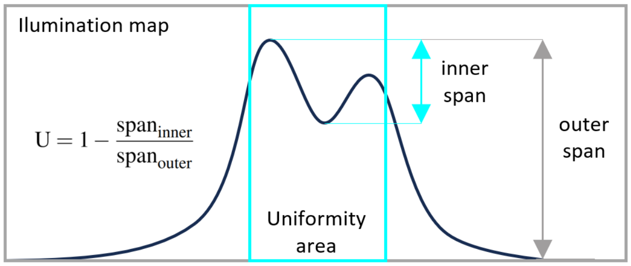

Figure S6. **Illumination uniformity** is computed from the ratio of the min-max span inside the "uniformity area" and the min-max span of a whole field i.e. zero to maximal amplitude/intensity of the field. Despite showing a one dimensional model, a 2D version is calculated in *ptychoScopy*. The "uniformity area" corresponds to the cyan square shown in Figure 3d, k, i.e. area without influence of border beam positions.

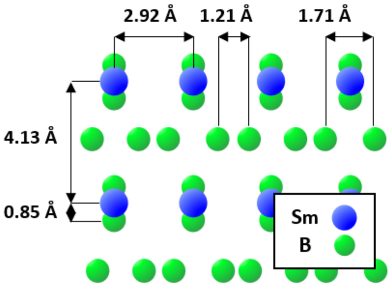

Figure S7. **Test sample.** Atomic arrangement of the SmB_6 crystal in [110] orientation.

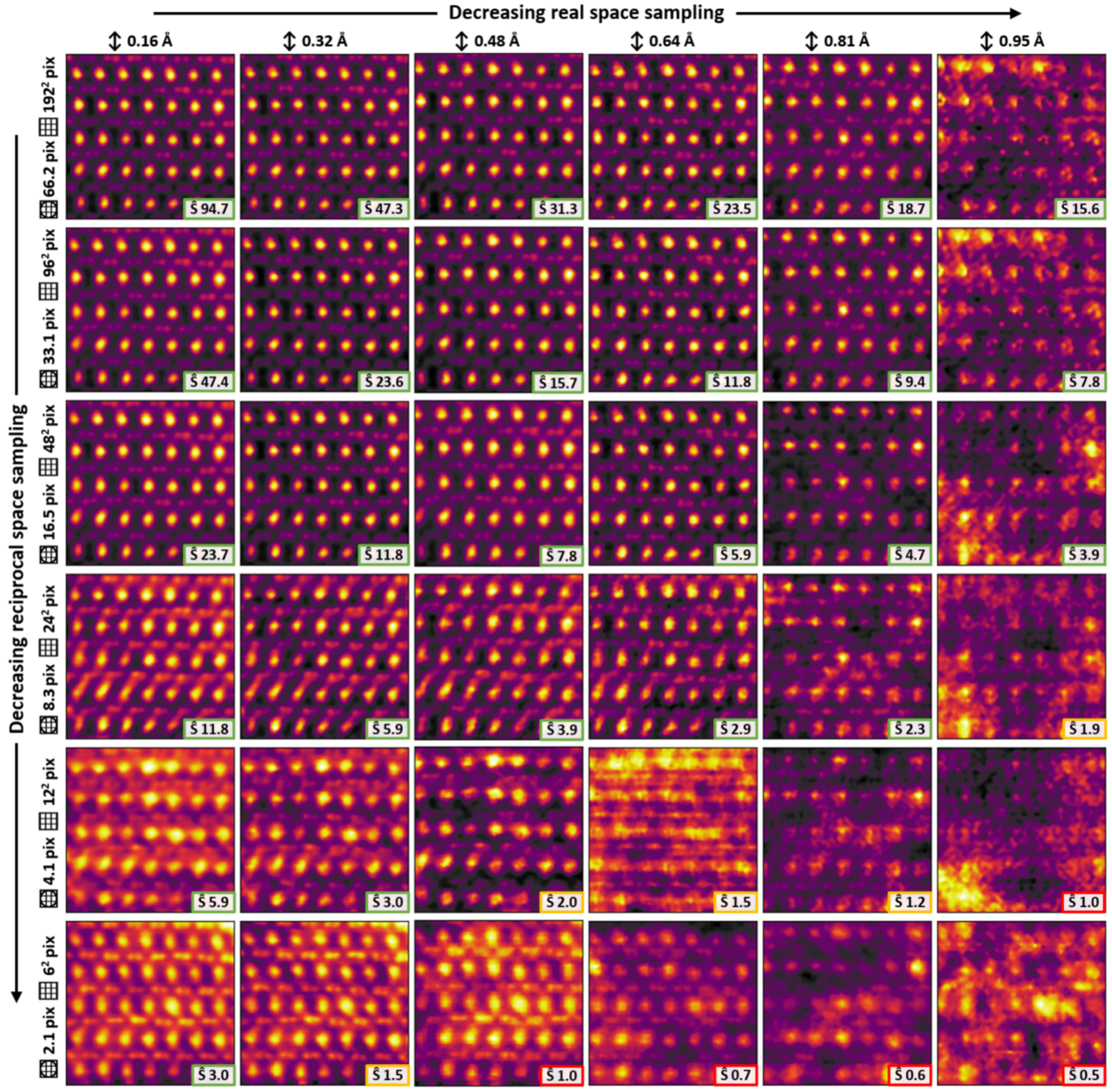

Figure S8. Combined real- and reciprocal-sampling reduction for singleslice ITR and its influence to reconstruction quality. The real space of the specimen covers an area of 2 nm×2 nm. Combined sampling ($\widehat{S}$) is shown in boxes and for qualitative assessment colour coded as follows $\widehat{S}$<1<$\widehat{S}$<2<$\widehat{S}$.
